# 3D-tips: user-friendly mesh barrier pipette tips for 3D-spheroid culture

**DOI:** 10.1186/s13036-019-0210-3

**Published:** 2019-10-28

**Authors:** Mo Chen, Marie-Laure Vial, James A. St John

**Affiliations:** 1 Clem Jones Centre for Neurobiology and Stem Cell Research, Griffith University, Brisbane, QLD 4111 Australia; 2 Griffith Institute for Drug Discovery, Griffith University, Brisbane, QLD 4111 Australia; 3 Menzies Health Institute Queensland, Griffith University, Southport, QLD 4222 Australia

**Keywords:** Three-dimensional cell culture, Spheroid, Drug screening, Immunofluorescence, Pipette tips

## Abstract

**Background:**

Three dimensional (3D) cell cultures have been an area of increasing interest and relevance across several research fields including drug discovery, developmental biology and stem cell-based therapies. However, handling 3D structures can be difficult. In particular, the replacement of liquid media and reagents in which liquid is removed using pipettes is difficult to perform as the 3D spheroids can be easily aspirated into the pipette tip.

**Results:**

We have developed the 3D-tip, a novel tool that facilitates media change and washing procedures of 3D-spheroid cultures. The 3D-tip contains a mesh with 40-μm pores allowing the aspiration of liquids including media, drugs, buffers and reagents, with the mesh acting as a barrier preventing the spheroids being aspirated into the pipette tip. After aspiration of liquids, the spheroids are gently deposited back into the culture vessel. Our results demonstrate that the 3D-tips offer superior handling of 3D-spheroid cultures in comparison to commonly used methods. We showed that the 3D-tips can easily be used on both fixed and unfixed spheroids and on cancer cell, stem cell and glial cell spheroids.

In contrast with the 50/50 media exchange method, the 3D-tips allow a complete media change with minimal loss of spheroids and without damaging their morphology. Our results showed that 86.0% of spheroids remained in the chamber after changing the media using the 3D-tips. In contrast, only 45.0% of spheroids remained using the 50/50 media exchange strategy.

In comparison with the centrifugation technique, the 3D-tips preserved spheroids whereas centrifugation led to the loss of spheroids and/or the alteration of the size and shape of the 3D cellular structures. We observed that 87.6 and 84.6% of the fixed and unfixed spheroids remained using the 3D-tip, respectively. In contrast, only 66.3% of the fixed spheroids and 36.4% of the unfixed spheroids were left using the centrifugation method. From a time perspective, the 3D-tips dramatically reduce the time taken for replacing media.

**Conclusions:**

This novel pipette tip is suitable for high throughput screening and automation and will revolutionise the techniques used for the production and analysis of 3D spheroids.

## Introduction

Three dimensional (3D) cell culture has led a revolution in eukaryotic cell culture. Cells grown in 3D can form more complex cell-cell interactions, which more closely mimic the in vivo situation than two dimensional cell culture [1]. Therefore this technology is booming in many fields, including cancer and stem cell research, drug screening and tissue engineering [2]. However, the production and use of 3D cell cultures with standard cell analysis techniques, which were originally developed for 2D cell culture systems, can be challenging. For instance, tasks including cell culture media change, drug testing or cell imaging techniques such as immunofluorescence staining, which require a large number of wash steps can be difficult to achieve when using 3D cell culture models. Due to the nature of the cell growth of 3D cell cultures, the spheroids or other 3D cell structures, tend to have low adherence to the vessel surface and thus during liquid changes the 3D structures can be easily aspirated. Methods commonly used for a media change or wash steps using 3D cell cultures currently include 50/50 media exchange [3], centrifugation [4] and free settling [5] techniques. However, these techniques are laborious, time-consuming, may damage the morphology of the spheroids or even lead to the loss of the spheroids, which are aspirated into the pipette tip. Hence, the need of developing a novel tool to easily perform media change and wash steps of 3D cell cultures.

Here, we describe the development of the 3D-tip for 3D cell culture studies. The 3D-tip is composed of a tip containing a mesh with 40-μm pores allowing the aspiration of media, buffers, reagents and drugs without losing or damaging the shape or size of the 3D multicellular spheroids. The 3D-tip can revolution the use of 3D cell cultures in several fields of research including cell biology, developmental biology and drug discovery.

## Results

### The 3D-tip is suitable for liquid handling with live (unfixed) 3D cell spheroids

**Fig. 1:**
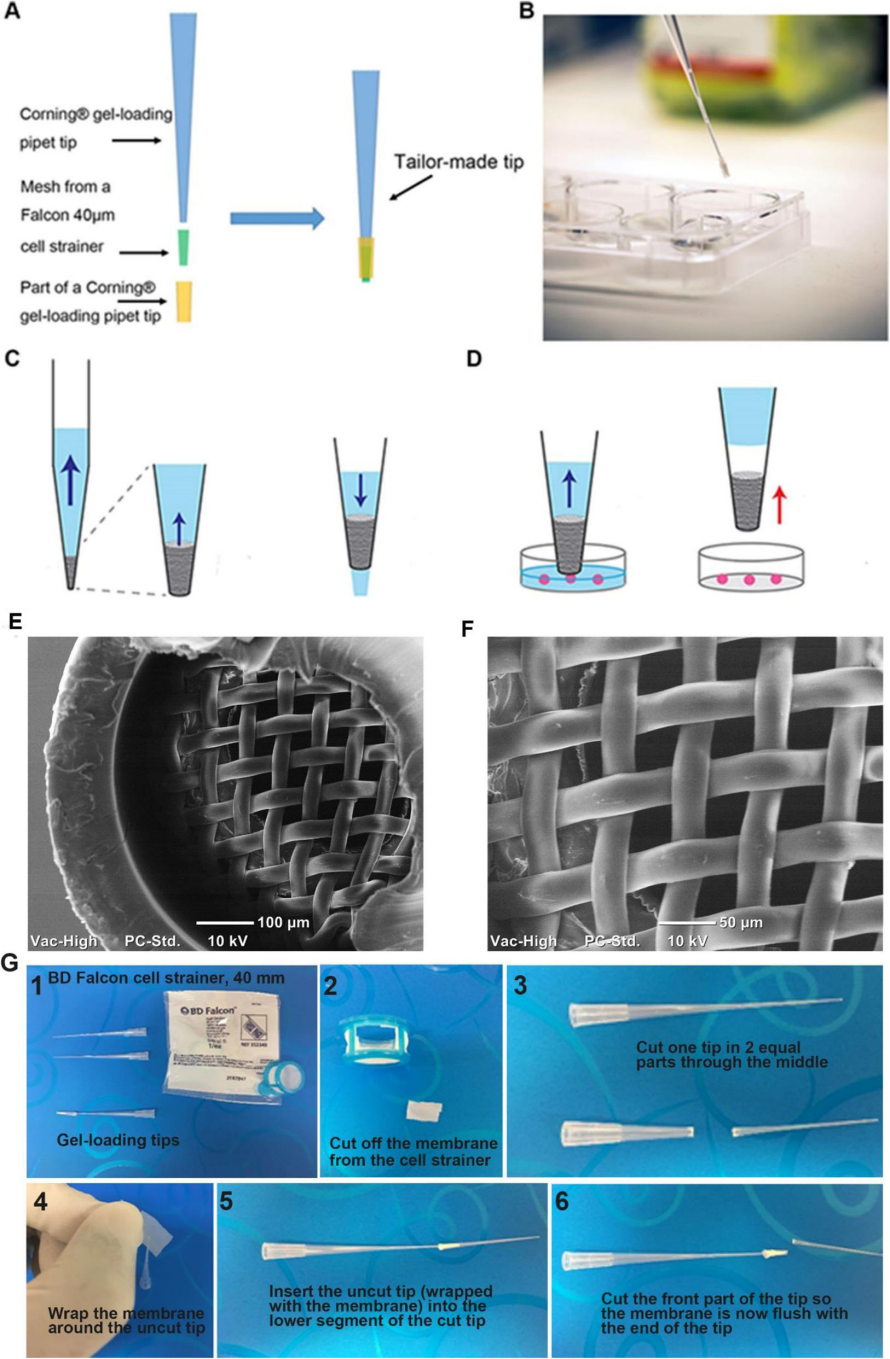
The use of 3D-tips to replace liquid in cell culture plates. **a** Assembly of the 3D-tip prototype. **b** Representative image of a 3D-tip. **c** A mesh barrier at the narrow end of the 3D- tip allowed rapid fluid flow in both directions. **d** Mesh barrier prevents small spheroids being aspirated into the pipette tip. Spheroids that are displaced during removal of the liquid are deposited back into the culture vessel. **e** Scanning electron microscope (SEM) image of a tip containing a mesh with 40-μm pores **f** SEM image of a mesh with 40-μm pores. **g** Step-by-step illustrative diagram describing the assembly of a 3D-tip

Three-dimensional cell cultures of olfactory ensheathing cells (OECs) were generated in naked liquid marbles (NLMs) [6] and then transferred into an 8-well chamber. Removal of the liquid medium is needed for several different processes including refreshing culture medium, adding specific reagents for assays, and replacing with fixative solution. To test the suitability of using the 3D-tips (Fig. 1a and b), the medium was removed from the chamber, which contained different sized spheroids. Large spheroids did not tend to be displaced during the removal of the medium. In contrast, small spheroids were displaced with the medium but were prevented from entering the pipette tip due to the mesh barrier and the spheroids subsequently remained in the culture vessel (Fig. 1c-g).

**Fig. 2:**
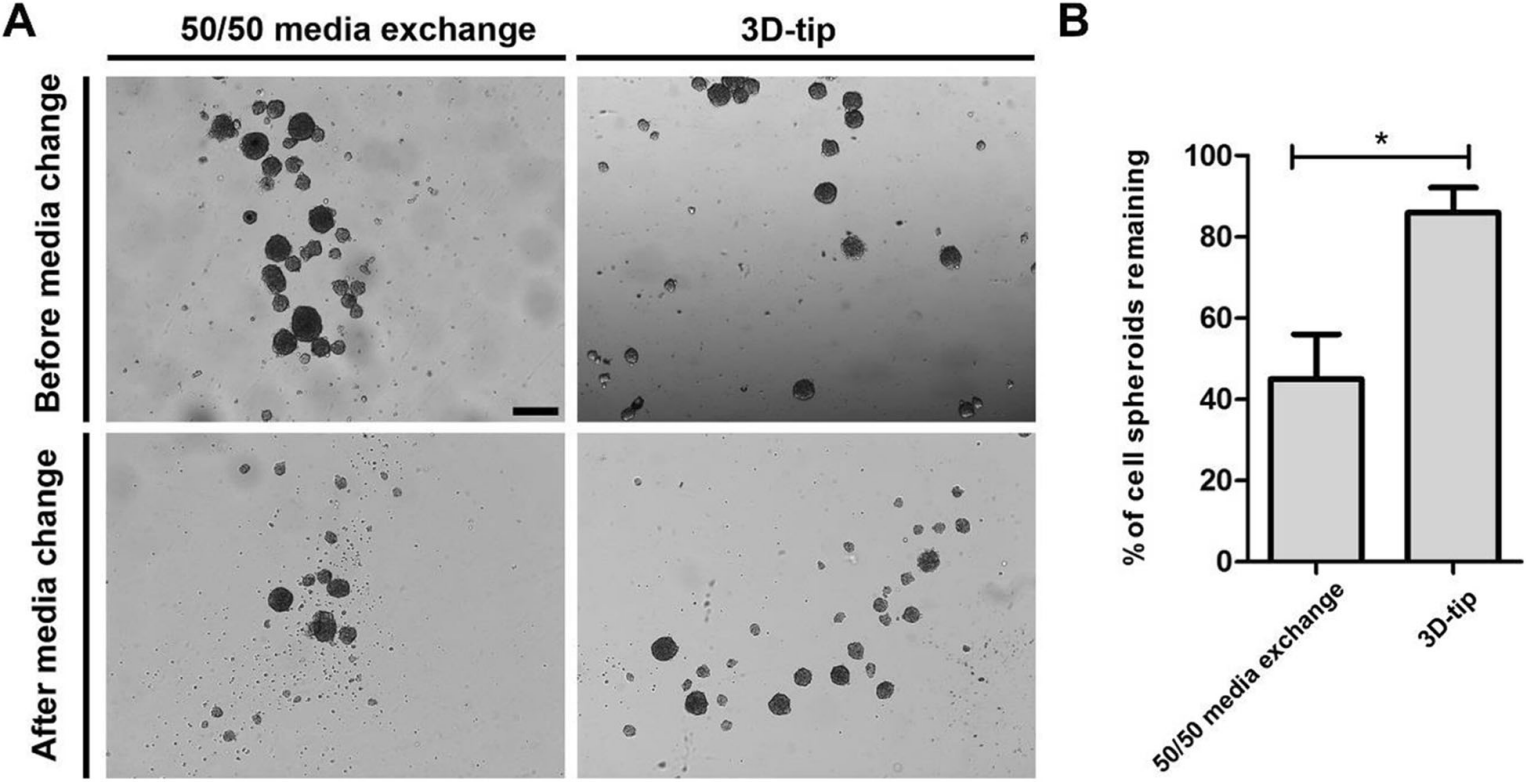
Comparative study between the 3D-tip system and the 50/50 media exchange method to change media of 3D cell cultures of OECs. **a** Representative images of 3D OEC spheroids before and after media change using the 3D-tip system and the 50/50 media exchange method. Scale bar = 200 μm. Images were captured on an Olympus IX73. **b** Percentage of spheroids remaining after media change using the 3D-tip system and the 50/50 media exchange method. P*< 0.05, *n* = 3, technical replicates

A comparative study between the 3D-tip system and the commonly used 50/50 media exchange method with normal tips was performed. First, the 3D-tip method allowed nearly a full media change while only half of the media was replaced with the 50/50 media exchange method. In addition, we observed that on average 86.0% of spheroids remained in the chamber after changing the media using the 3D-tips (Fig. 2a and b). In contrast, only 45.0% of spheroids remained using the 50/50 media exchange method showing that most of spheroids were aspirated and lost using this strategy (Fig. 2a and b). Consequently, the percentage of spheroids lost during the media change procedure using the 3D-tips significantly decreased in comparison with the 50/50 media exchange method using normal tips (*p* = 0.0158). As there was also some residual media left in the wells after the media exchange using the 3D-tips, smaller aggregates of cells were also retained within the well. No bacterial contamination was observed after changing the media using the 3D-tip.

**Fig. 3:**
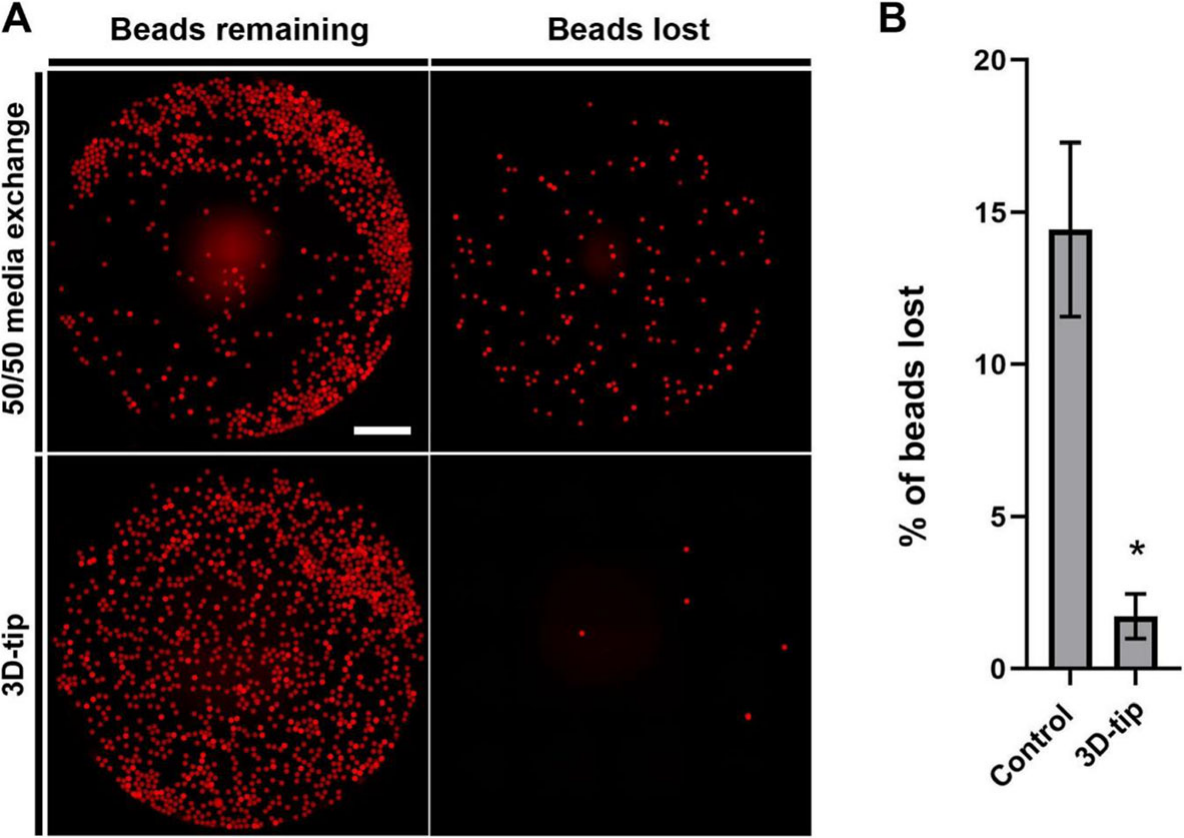
Comparative study between the 3D-tip system and the 50/50 media exchange method to change media of latex beads from polystyrene (100 μm). **a** Representative images of latex beads after media change using the 3D-tip system and the 50/50 media exchange method in 96-well plate. Left panels show the latex beads that remained in the well. Right panels show the latex beads lost during the media change. Scale bar = 1000 μm. **b** Percentage of latex beads lost after media change using the 3D-tip system and the 50/50 media exchange method. P*< 0.05, *n* = 3, technical replicates

Latex beads from polystyrene (100 μm) were used to assess the proportion of beads lost after media change using 3D-tips and the 50/50 media exchange method. We observed that on average approximately 14% of beads were aspirated and lost after changing the media using the 50/50 media exchange method (Fig. 3a and b). In contrast, only 2% of beads were lost using the 3D-tips showing that most of spheroids remained in the well using this strategy (Fig. 3a and b). Consequently, the percentage of beads lost during the media change procedure using the 3D-tips significantly decreased in comparison with the 50/50 media exchange method using normal tips (*p* < 0.05) (Fig. 3b).

### The 3D-tip is suitable for immunofluorescence microscopy using fixed 3D cell spheroids

**Fig. 4:**
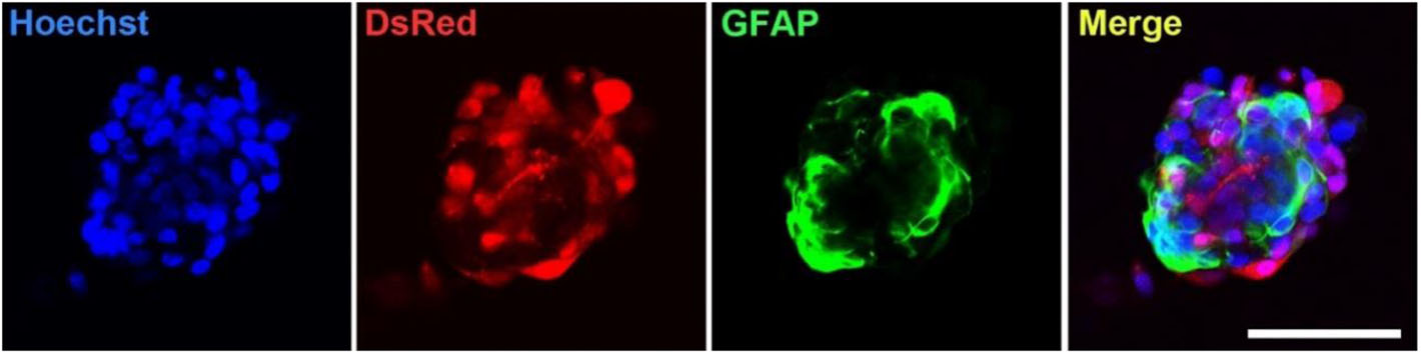
Immunofluorescence assay of 3D co-cultures using the 3D-tip. Co-culture of astrocytes detected by anti-GFAP (green) and S100β-DsRed Schwann cells (red). Cells were stained with Hoechst (blue). Scale bar = 100 μm. Images were captured on an Olympus FV1000 confocal microscope

To determine whether the 3D-tip is suitable for handling 3D spheroids during immunofluorescence assays, we performed 3D co-cultures of astrocytes with S100β-DsRed Schwann cells (SC) (Fig. 4) in NLMs. After fixation of the cell spheroids, immunofluorescence assays were performed using the 3D-tips for all liquid changes. Anti-glial fibrillary acidic protein (GFAP) antibodies were used to detect astrocytes, and Hoechst was used to stain nucleic acids to visualize nuclei. We observed a specific detection of GFAP in astrocytes, and a specific detection of nuclei by Hoechst stain as well as a low background signal. The accurate immunofluorescence and nuclear staining, together with the intact morphology of the 3D cell structures, show that the 3D-tips are suitable for performing the assay and wash steps during immunofluorescence assays without damaging the spheroids.

**Fig. 5:**
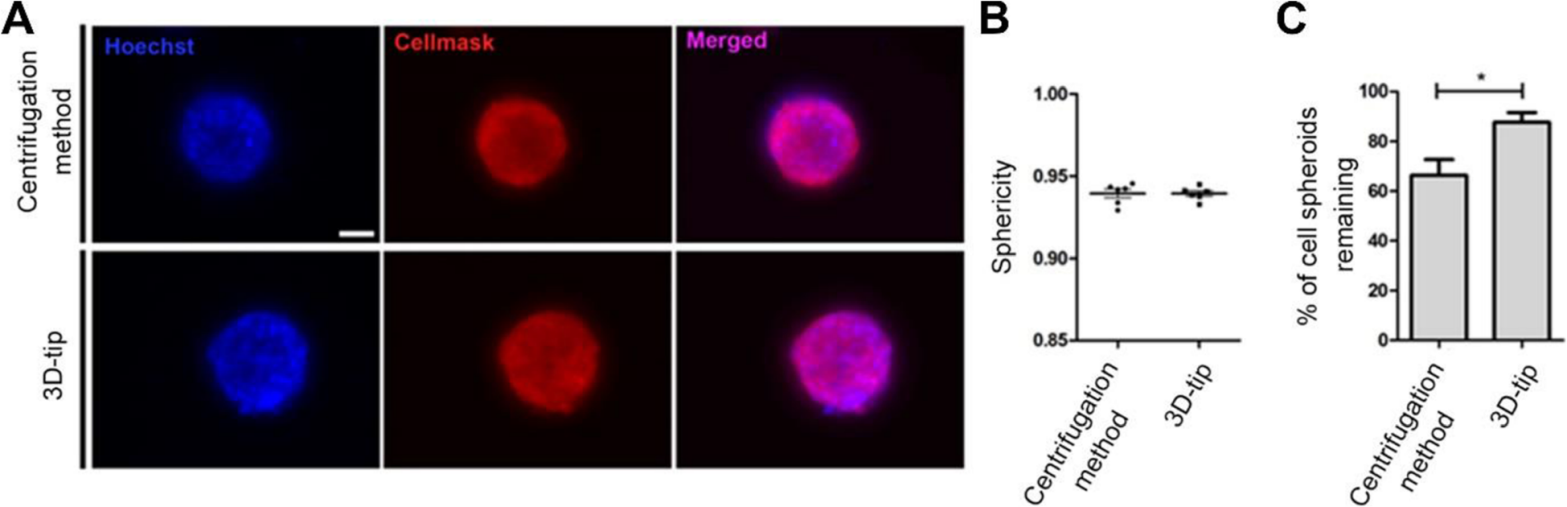
Comparison between the 3D-tip and the centrifugation methods for fluorescence staining of fixed 3D OEC cultures. **a** Representative images of fixed 3D OEC spheroids stained with Hoechst (blue) and CellMask Deep Red (red) using the 3D-tip and the centrifugation methods. **b** Sphericity analysis of 3D structures after fluorescence staining using the 3D-tip and the centrifugation methods. **c** Percentage of spheroids remaining after fluorescence staining using the 3D-tip and the centrifugation methods. Scale bar = 50 μm. Images were captured on an Olympus IX73 microscope. The analysis was performed using AnaSP 1.0. p*< 0.05, *n* = 3, technical replicates

A comparative study between the 3D-tip system and the commonly used centrifugation method for fluorescence microscopy was then performed in parallel using fixed 3D cell spheroids. The size of spheroids ranged from 41 to 148 μm in diameter with an average size of 78 μm in diameter (SEM = 2.52 μm). We observed that the sphericity of the 3D structures was maintained with both methods (Fig. 5a and b). However, 87.6% of spheroids remained using the 3D-tip while, in contrast, only 66.3% of spheroids were left using the centrifugation method (Fig. 5c). Consequently, the percentage of spheroids retained using the 3D-tips significantly increased in comparison with the centrifugation method (*p* = 0.0234). In addition, the centrifugation step with three washes required 20 min whereas the use of the 3D-tip required less than 1 min for the wash steps. Thus, the use of the 3D-tip reduces the time needed for assays.

In addition, no clogging of the pores was observed during the multiple washes for the different staining protocols.

### Comparative study between the 3D-tip method and the centrifugation technique for fluorescence labelling of live (unfixed) 3D cell spheroids

**Fig. 6:**
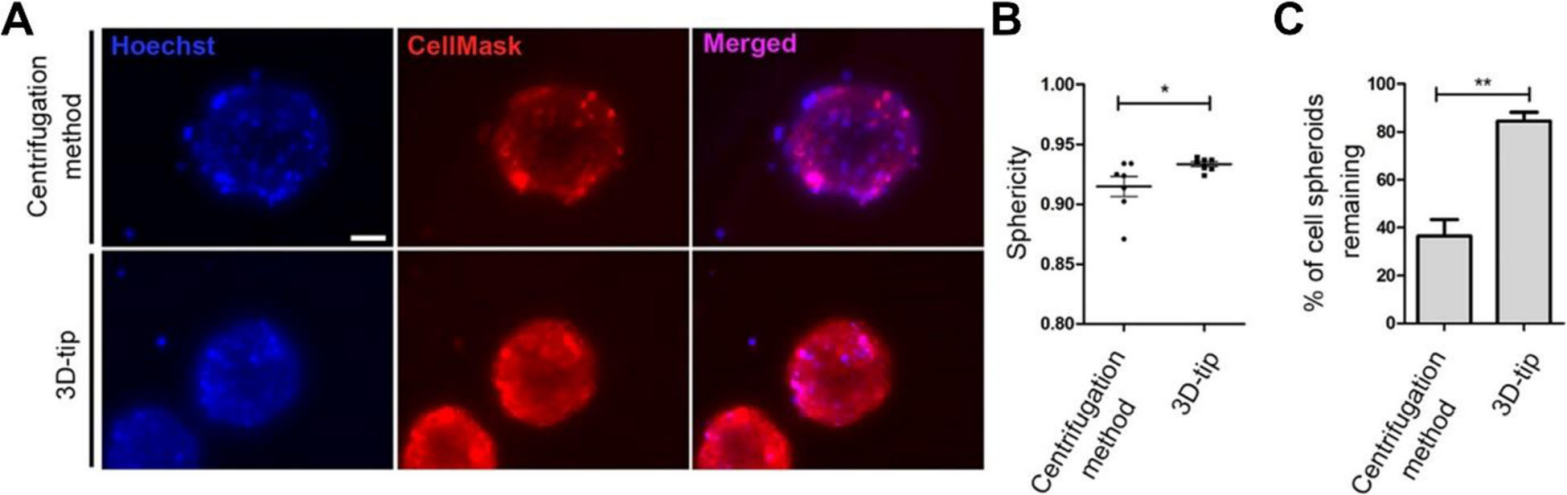
Comparison between the 3D-tip and the centrifugation methods for fluorescence labelling of live (unfixed) 3D OEC cultures. **a** Representative images of unfixed 3D OEC spheroids stained with Hoechst (blue) and CellMask Deep Red (red) using the 3D-tip and the centrifugation methods. **b** Sphericity analysis of 3D structures after the staining assay using the 3D-tip and the centrifugation methods. **c** Percentage of spheroids remaining after the staining assay using the 3D-tip and the centrifugation methods. Scale bar = 50 μm. Images were captured on an Olympus IX73 microscope. The analysis was performed using AnaSP 1.0. p*< 0.05, p**< 0.01, *n* = 3, technical replicates

Labelling of live cells is often routinely performed with a range of different stains. We therefore performed a comparative study between the 3D-tip system and the commonly used centrifugation method for staining live (unfixed) cell spheroids. We observed that the sphericity of the spheroids significantly decreased with the centrifugation method (0.91) compared to the 3D-tip method (0.93) (*p* = 0.0265) (Fig. 6a and b). In addition, 84.6% of spheroids remained using the 3D-tip while, in contrast, only 36.4% of spheroids were left using the centrifugation method (Fig. 6c). Consequently, the percentage of spheroids retained using the 3D-tips significantly increased in comparison with the centrifugation method (*p* = 0.0018).

### The 3D-tip is suitable for various cell types grown in 3D cell culture

**Fig. 7:**
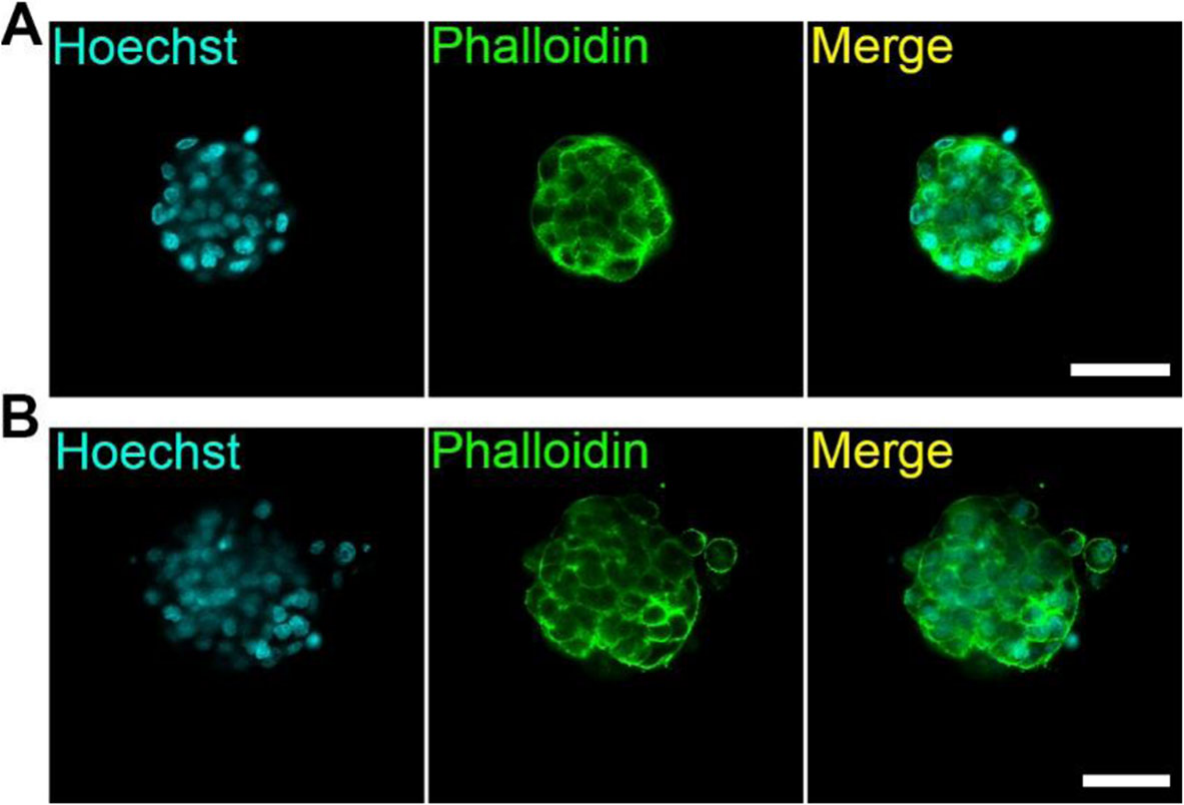
Fluorescence staining of 3D cell cultures of BxPC-3 and ReNcell VM using the 3D-tip system. **a** F-actin arrangement of 3D cultures of BxPC-3 cells detected by Alexa Fluor™ 488 Phalloidin (green). **b** F-actin arrangement of 3D cultures of ReNcell VM detected by Alexa Fluor™ 488 Phalloidin (green). Nuclei were visualized with Hoechst (blue). Scale bar = 50 μm. Images were captured on an Olympus FV3000 confocal microscope

3D cultures of pancreatic cancer cells (BxPC-3) and neural progenitor cells (ReNcell VM) were used to determine whether the 3D-tips were suitable for various cell types. BxPC-3 and ReNcell VM spheroids were fixed and stained with Alexa Fluor™ 488 Phalloidin to visualize actin filaments (F-actin) and Hoechst to stain the nuclei. All the washes were performed using 3D tips. A regular F-actin arrangement around the nucleus was observed (Fig. 7a and b). Therefore, 3D-tips can be used on different cells types.

### The 3D-tip does not generate long-term effects on cell viability

**Fig. 8:**
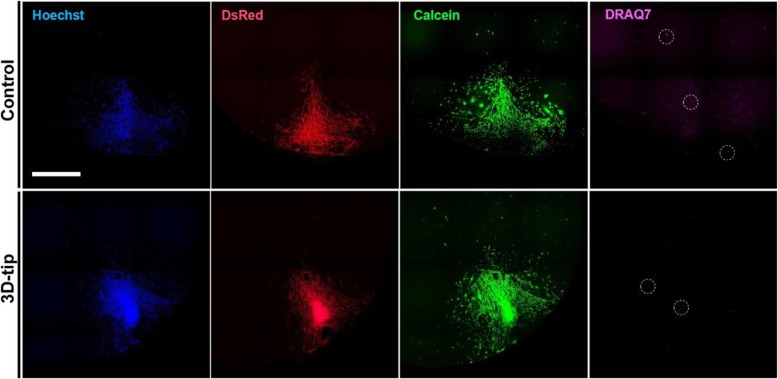
Long-term effects of the 3D-tip method on cell viability. The culture medium of primary S100β-DsRed OECs spheroids was changed using the 3D-tip method or the 50/50 media exchange (control) method. After 4-day incubation, primary S100β-DsRed OECs (red) migrating out from the spheroids were stained with Hoechst (blue) to visualize nuclei along with the cell-permeant dye Calcein AM (green) to determine cell viability and DRAQ7 dye (pink), which stains dead cells. Scale bar = 1000 μm. *n* = 2. Images were captured on a Nikon Ti2-E inverted microscope

To determine whether the 3D-tips have long-term effects on cell viability, we used 3D spheroids of primary S100β-DsRed OECs generated in the NLM system. The spheroids were then transferred into a 96-well plate. Media change was either performed using the 3D-tips or normal tips (50/50 media exchange, control). After 4-day incubation, the cells were stained with Hoechst to visualize nuclei along with the cell-permeant dye Calcein AM to determine cell viability and DRAQ7 dye, which stains dead cells. Our results demonstrate that with both the 3D-tip method and the 50/50 media exchange method (control), cells migrate out from the spheroid (Fig. 8). The majority of the cells were Calcein-positive and only a few cells were positive for DRAQ7 showing that the 3D-tips do not affect the viability of primary S100β-DsRed OECs (Fig. 7). In addition, no bacterial contamination was observed (Fig. 8).

## Conclusion

Studies have shown that three-dimensional (3D) cell cultures are more reflective of the in vivo state. However, standard cell analysis methods developed for 2D cell cultures are not always adapted to 3D cellular models. Indeed, most of commonly used methods for media change or washing procedures using 3D multicellular spheroids are often time-consuming and can damage the cells and alter the morphology of the 3D structures, seriously affecting the experimental outcomes.

Our results demonstrate that the novel 3D-tips offer superior handling of 3D cell cultures in comparison to other commonly used methods and allow more rapid media exchange with increased retention of 3D spheroids, without causing damage to the gross morphology. In contrast with the 50/50 media exchange method, the 3D-tips allow a complete media change with minimal loss of the spheroids and without damaging the morphology of the spheroids. In comparison with the centrifugation technique, the 3D-tips preserved spheroids whereas centrifugation led to the loss of spheroids and/or the alteration of the size and shape of the 3D cellular structures.

Even though, the time and cost of the 3D-tip assembly is slightly higher than the normal tips (Additional file 1: Table S1), the 3D-tip has been designed to overcome many limitations of handling 3D cell cultures, and particularly targets the need to reduce time needed for media exchange and, reduce the volume of media needed for wash steps, and to improve the retention and morphology of the spheroids during handling steps. Its mesh with pores as small as 40 μm allows a complete media change and facilitates the washing steps during immunofluorescence staining, with reduced loss of the spheroids and with minimal damage. The 3D-tips are assembled using aseptic techniques. Every component of the tip, including the Corning gel-loading pipette tip and the mesh from a Falcon 40 μm cell strainer, is sterile. The 3D-tips are assembled in a biosafety cabinet, handled using gloves sprayed with ethanol and kept sterile until they are used for experiments. In addition, the assessment of long-term effects of the 3D-tips on cell viability did not show any bacterial contamination (Fig. 8).

From a cost perspective, particularly for high-throughput screening, a 50/50 media change significantly increases the cost of reagents and therefore the 3D-tip will reduce costs of the assays. In addition, the 3D-tip is cost-effective because of labour-saving. The ability to remove all the media in one easy step using the 3D-tip is particularly useful for drug screening assays when it is crucial to completely remove the media. We showed that the 3D-tips can easily be used on both fixed and unfixed spheroids and on different cell types including cancer cell, stem cell and glial cell spheroids. From a time perspective when compared to the centrifugation method, the 3D-tips dramatically reduce the time taken for replacing media.

Taken together, the results demonstrate that the 3D-tips satisfy the unmet need for ease of handling liquids for 3D cell culturing and immunofluorescence assays using 3D multicellular spheroids. This tool is also suitable for washing out drugs during drug screenings and could be used in association with automated liquid handling platforms.

## Methods

### Cell culture conditions

Immortalized mouse OECs were generated from olfactory bulb ensheathing glia of GFP-expressing mice (C57BL/6-Tg (ACTB-EGFP)1Osb/J, Jackson Laboratory, Bar Harbor, USA [7, 8]. mOEC-GFP were cultured in Dulbecco’s Modified Eagle Medium/Nutrient F-12 (DMEM/F12) supplemented with 10% fetal bovine serum (FBS, Bovogen) and 10 ng/mL gentamicin (Life Technologies). Dorsal root ganglion Schwann cells and astrocytes were isolated from S100β-DsRed transgenic mice [9]. Schwann cells and astrocytes were maintained in complete medium conditions (Dulbecco’s Modified Eagle Medium (DMEM) supplemented with 10% FBS, 1% GlutaMAX™ (Life Technologies) supplement and 50 ng/mL gentamicin). Pancreatic cancer cell (BxPC-3) and Neural stem cells (ReNcell VM) were maintained in DMEM supplemented with 10% FBS and 10 ng/mL gentamicin and in DMEM/F12 supplemented with 10% fetal bovine serum, 1% N-2 Supplement (Life Technologies) and 10 ng/mL gentamicin, respectively. All the cells were maintained in a humidified incubator at 37 °C and 5% CO2.

### 3D-tip assembly

Due to limitations in our ability to manufacture the 3D-tips, a prototype was assembled in several steps. First, the mesh of a Falcon 40 μm cell strainer (#352340) was cut. The mesh was approximately 30 mm wide and 80 mm long. The Corning® gel-loading pipet tip (#CLS4884) was cut at a length of approximately 300 mm. The 80 mm × 30 mm mesh was then wrapped around the point of an intact Corning® gel-loading pipet tip (#CLS4884) and inserted inside the Corning® gel-loading pipette tip (#CLS4884) previously cut. Finally, the point of the tip was trimmed and the 3D-tip was then ready for use (Fig. 1).

### Sputter coating and SEM analysis

Dried 3D tips were cut and glued to the specimen stubs by carbon adhesive discs, then gold (Au) sputter coated in a in a Jeol MP-19020NCTR. The mesh was then examined with a JCM-5000 NeoScope™ Table Top SEM.

### Generation of spheroids using the NLM system

3D cultures were produced using the NLM system that we have developed (Australian provisional patent 2017904456 [6];). In this system, 384-well, PS, F-Bottom, Small volume, HiBase microplates (#784075, Grenier Bio-One) were used to generate coated plates with the NeverWet Multi-Purpose Spray Kit (Rust Oleum). The superhydrophobic-coated plates were used for NLM generation. This system enables cells to freely associate to form highly reproducible spheroids.

Cells were harvested and manually counted with a haemocytometer and seeded in superhydrophobic-coated plates at specified densities using complete medium conditions. Spheroids were then collected after 48 or 72 h and used for subsequent experiments.

Provisional patent details: Chen M, Vial ML, Tello Velasquez J, Barker M, St John JA. Naked liquid marbles: a robust three-dimensional low-volume cell culturing system. Provisional patent filed 2 November 2017, number 2017904456, Australian Government IP Australia.

### Complete media change using the 3D-tip

Cell spheroids were transferred into an 8-well chamber containing 200 μL of medium. The medium was aspirated using the 3D-tip and 200 μL of fresh medium was added. Spheroids were imaged before and after media change using the Olympus IX73 and image analysis was performed using the software cellSens. The spheroids were maintained in culture for 7 days without media change for contamination assessment.

### 50/50 media exchange

Half of the medium (100 μL) was carefully removed using a P200 pipette and a normal tip. One hundred microliters of fresh medium was added into each well. Spheroids were imaged before and after media change using the Olympus IX73 and image analysis was performed using the software cellSens.

### Live (unfixed) 3D cell spheroid fluorescence staining using the 3D-tip

3D spheroids were then stained with Hoechst (1:2000) and CellMask Deep Red (1:2000) in culture media for 20 min at 37 °C, 5% CO_2_. Spheroids were washed three times with PBS using the 3D-tip and then stored in the dark at 4 °C. Spheroids were imaged using the Olympus IX73 after staining. The number of cell spheroids was accounted before and after staining.

### Live (unfixed) 3D cell spheroid fluorescence staining using the centrifugation method

3D OEC spheroids were stained with Hoechst (1:2000) and CellMask Deep Red (1:2000) in culture media for 20 min at 37 °C, 5% CO_2_. The spheroids were then transferred into a 1.5 ml Eppendorf tube, centrifuged for 5 min at 1000 rpm and the supernatant was aspirated using a P200 pipette and a normal tip. Spheroids were washed three times with PBS using the centrifugation method (5 min at 1000 rpm) as previously and then stored in the dark at 4 °C. Spheroids were imaged using the Olympus IX73 after staining. The number of cell spheroids was determined before and after staining.

### Fixed 3D cell spheroid fluorescence staining using the 3D-tip

3D OEC spheroids were fixed using 3% PFA for 24 h at 4 °C followed by three washes with PBS using a P200 pipette and the 3D-tip. 3D spheroids were then stained with Hoechst (1:2000) and CellMask Deep Red (1:2000) in PBS for 20 min at RT with continuous shaking. Spheroids were washed three times with PBS using the 3D-tip and then stored in the dark at 4 °C. Spheroids were imaged using the Olympus IX73.

BxPC-3 and ReNcell spheroids were fixed using 3% PFA for 24 h at 4 °C, then permeabilised by 0.3% Triton-X 100 for 60 min at RT with continuous shaking. Then spheroids were stained with Alexa Fluor™ 488 Phalloidin (1:1600) in PBS for 60 min at RT with continuous shaking. Spheroids were washed three times with PBS using the 3D-tip and then stored in the dark at 4 °C. Spheroids were imaged using the Olympus FV3000 confocal laser scanning microscope.

### Fixed 3D cell spheroid fluorescence staining using the centrifugation method

3D OEC spheroids were fixed using 3% PFA for 24 h at 4 °C. The spheroids were then transferred into a 1.5 ml Eppendorf tube, centrifuged for 5 min at 1000 rpm and the supernatant was aspirated using a P200 pipette and a normal tip. 3D cell spheroids were washed three times with PBS using the centrifugation method (5 min at 1000 rpm) as previously described. Spheroids were then stained with Hoechst (1:2000) and CellMask Deep Red (1:2000) in PBS for 20 min at RT with continuous shaking. The spheroids were centrifuged for 5 min at 1000 rpm and the supernatant was aspirated using a P200 pipette and a normal tip. Spheroids were washed three times with PBS using the centrifugation method (5 min at 1000 rpm) as previously and then stored in the dark at 4 °C. Spheroids were imaged using the Olympus IX73 after staining. The number of cell spheroids was determined before and after staining.

### Immunocytochemistry using 3D co-cultures

For immunofluorescence microscopy studies, the medium was aspirated using a P200 pipette and the 3D-tip and 3D co-cultures of astrocytes with SC were fixed in 3% PFA for 24 h at 4 °C before being washed three times with PBS using a P200 pipette and the 3D-tip. Spheroids were then treated with 0.2% Triton X-100 and 2% bovine serum albumin (BSA) for 1 h at RT and washed three times with PBS. PBS was aspirated and goat anti-GFAP antibodies (1:500 in PBS) was added and the 8-well chamber was incubated at RT for 3 h with continuous shaking. Spheroids were washed three times with PBS using the 3D-tip. Secondary antibody Alexa Fluor® 488 Donkey anti-goat (1:500 in PBS) was added for 1 h at RT with continuous shaking. 3D spheroids were washed three times with PBS and stained with Hoechst (1:2000) for 15 min at RT. Spheroids were washed three times with PBS and stored in the dark at 4 °C. Spheroids were imaged using the Olympus FV1000 confocal microscope.

### Live/dead cell viability assay

Primary S100β-DsRed OEC spheroids were generated in the NLM system at a cell seeding density of 100,000 cells/well in a 384-well plate. After 48 h, the spheroids (~ 500 μm diameter) were transferred into a 96-well plate. The media was changed using the 3D-tips or the 50/50 media exchange method (standard tips (Vetex, 200 μL)). After 96 h incubation, the cells were stained with Hoechst (1:2000, Thermo Fisher Scientific) to visualize nuclei along with the cell-permeant dye Calcein AM (1 μM, Thermo Fisher Scientific, Catalogue number: C3100MP) to determine cell viability and DRAQ7 dye (1:500, Abcam), which stains the dead cells, for 15 min. Images were captured using a Nikon Ti2-E microscope. The experiment was performed in duplicate.

### Media change using latex beads from polystyrene

Latex beads from polystyrene (100 μm, Sigma-Aldrich) were used to assess the proportion of beads lost after media change using the 3D-tips and the 50/50 media exchange method. Approximately 600 beads were transferred into a 96-well plate containing cell culture media. Media change was performed using the 3D-tips or the 50/50 media exchange method. The medium aspirated was transferred into another well. Images of beads pre and post media change were captured using a Nikon Ti22-E microscope. The number of beads was determined using the open-source imaging analysis software Cell profiler 3.1.8. The experiment was performed in triplicate.

## Supplementary information

**Additional file 1: Table S1.** Quantitative comparison between the 3D-tip method, the 50/50 media exchange method (normal tip) and the centrifugation method.

## Data Availability

The data generated and analysed during this study is available from the corresponding author on reasonable request.
