# Supplementary material for: 3D-tips: user-friendly mesh barrier pipette tips for 3D-spheroid culture
Source: J Biol Eng. 2019 Oct 28;13:80. doi: 10.1186/s13036-019-0210-3 (PMC6819412; doi:10.1186/s13036-019-0210-3)
Supplement: Supplementary file 1 — Additional file 1: Table S1. Quantitative comparison between the 3D-tip method, the 50/50 media exchange method (normal tip) and the centrifugation method. [file 13036_2019_210_MOESM1_ESM.docx]

Table S1 Quantitative comparison between the 3D-tip method, the 50/50 media exchange method (normal tip) and the centrifugation method.
